# Dissecting the Role of N6-Methylandenosine-Related Long Non-coding RNAs Signature in Prognosis and Immune Microenvironment of Breast Cancer

**DOI:** 10.3389/fcell.2021.711859

**Published:** 2021-10-06

**Authors:** Jinguo Zhang, Benjie Shan, Lin Lin, Jie Dong, Qingqing Sun, Qiong Zhou, Jian Chen, Xinghua Han

**Affiliations:** Department of Medical Oncology, The First Affiliated Hospital of USTC, Division of Life Science and Medicine, University of Science and Technology of China, Hefei, China

**Keywords:** breast cancer, m6A-related LncRNAs, immune infiltration, gene signature, prognosis

## Abstract

Breast cancer (BC) represents a molecularly and clinically heterogeneous disease. Recent progress in immunotherapy has provided a glimmer of hope for several BC subtypes. The relationship between N6-methyladenosine (m6A) modification and long non-coding RNAs (LncRNAs) is still largely unexplored in BC. Here, with the intention to dissect the landscape of m6A-related lncRNAs and explore the immunotherapeutic value of the m6A-related lncRNA signature, we identified m6A-related lncRNAs by co-expression analysis from The Cancer Genome Atlas (TCGA) and stratified BC patients into different subgroups. Furthermore, we generated an m6A-related lncRNA prognostic signature. Four molecular subtypes were identified by consensus clustering. Cluster 3 preferentially had favorable prognosis, upregulated immune checkpoint expression, and high level of immune cell infiltration. Twenty-one m6A-related lncRNAs were applied to construct the m6A-related lncRNA model (m6A-LncRM). Survival analysis and receiver operating characteristic (ROC) curves further confirmed the prognostic value and prediction performance of m6A-LncRM. Finally, high- and low-risk BC subgroups displayed significantly different clinical features and immune cell infiltration status. Overall, our study systematically explored the prognostic value of the m6A-related LncRNAs and identified a high immunogenicity BC subtype. The proposed m6A-related LncRNA model might serve as a robust prognostic signature and attractive immunotherapeutic targets for BC treatment.

## Introduction

The Global Cancer Statistics reported that female breast cancer (BC) surpassed lung cancer as the most diagnosed cancer, with an estimated 2.3 million new cases (11.7%) (Sung et al., 2021). BC is generally regarded as a heterogeneous disease in terms of its molecular features, histological composition, and clinical characteristics (McCart Reed et al., 2021; Sadeghalvad et al., 2021). With the evolution of high-throughput technologies, we tend to subtype the disease into clinically relevant molecular subtypes, including normal-like, luminal A and B, HER2-enriched, and basal-like or more intrinsic subtypes (Ochoa et al., 2020; Morgan et al., 2021). The five clinically molecular subtypes have drastically different treatment selection, prognosis, and tumor biology (Zubair et al., 2020). The purpose of BC molecular subtyping is to design personalized treatment strategies for patients based on the emerging evidence of BC classification and treatment responses (Pashayan et al., 2020). However, the subtyping of BC remains unexplored and challenging, which manifests as a heterogeneity of therapeutic responses and prognosis within the same clinical subtypes.

N6-Methyladenosine (m6A) is the most abundant internal epigenetic modification occurring in RNA molecules (Wang et al., 2020). The discovery of m6A RNA modification has added a new layer of regulatory mechanism controlling gene expression (Nombela et al., 2021). Increasing evidence implicates that m6A regulators (“writers,” “erasers,” and “readers”) exert an essential role in multiple types of cancer by regulation of the “epi-transcriptome” in cancers (Huang et al., 2020; Liu et al., 2020). Remarkably, both m6A “writers” and “erasers” regulators are abnormally overexpressed and perform an oncogenic role in BC (Wei et al., 2020). The long non-coding RNAs (lncRNAs), a large class of conserved endogenous RNAs, are characterized as transcripts longer than 200 nucleotides and no protein-coding potential (Statello et al., 2021). m6A modification has been observed not only in messenger RNA but also in lncRNAs, affecting the fate of the modified RNA molecules (Lence et al., 2019). The antibody-based approach m6A-seq (MeRIP-seq) analysis has revealed that lncRNAs were also involved in such modification (He et al., 2020). Currently, m6A modification of long non-coding RNAs is still largely unexplored in BC.

N6-methyladenosine modification on RNA was involved in various stages of the RNA life cycle, including RNA transcription, processing, splicing, degradation, and RNA translation (Wei et al., 2020). m6A regulators act as either oncogenes or tumor suppressors to mediate the development and progression of BC. Aberrant expression of METTL3 and METTL14 has been reported to be associated with BC cell proliferation, migration, and metastasis (Wu et al., 2019; Shi et al., 2020). Demethylase FTO was highly expressed in BC, and upregulation of FTO enhanced the aggressiveness properties of BC, especially for HER2-overexpressing BC (Tan et al., 2015). Another m6A demethylase, ALKBH5, was recently reported to play a vital role in tumor formation and self-renewal of BC stem cells (Zhang et al., 2016a,b). The oncogenic role of “readers” proteins such as YTHDF1, YTHDF1, HNRNPC, HNRNPA2B1, and IGF2BP2 has been previously investigated in BC (Zheng et al., 2021). Regarding the association of m6A regulators and clinical features of BC, m6A regulators have been reported to significantly correlate with the clinicopathological characteristics, survival outcomes, and antitumor immune response in BC (He et al., 2021). Presently, increased attention was being paid to lncRNA signature models for its prognostic and predictive potential in cancer treatment (Li et al., 2019; Xu et al., 2021). Thus, dissecting the role of m6A-related lncRNA signature in BC may help researchers identify significant potential biomarkers for clinical applications. The emergence of immune checkpoint inhibitors (ICIs) has contributed to a revolutionary shift for traditional cancer treatment. Recent preclinical and clinical trials have supported that ICIs are also promising approaches in treating BC, particularly for triple-negative breast cancer (TNBC). PD-L1 checkpoint inhibitor atezolizumab in combination with nanoparticle albumin-bound (nab)-paclitaxel was approved in PD-L1-positive advanced or metastatic TNBC (Schmid et al., 2018). In the KEYNOTE-355 study, progression-free survival benefit was observed in pembrolizumab combined with chemotherapy compared with chemotherapy alone (Cortes et al., 2020). Depending on the results of the KEYNOTE-355 study, pembrolizumab combined with chemotherapy is currently FDA-approved for unresectable locally advanced or metastatic PD-L1-positive TNBC. In the present study, we aimed to depict the landscape of m6A-related lncRNAs and explore the immunotherapeutic value of the m6A-related lncRNA signature in the TCGA breast cancer (TCGA-BRCA) cohort. Furthermore, an m6A-related lncRNA prognostic model was constructed using least absolute shrinkage and selection operator (LASSO) regression. The role of immune infiltration and microenvironment heterogeneity in different BC subtypes and high-risk and low-risk subgroups was also investigated.

## Materials and Methods

### Data Processing of the Cancer Genome Atlas-Breast Cancer Dataset

A total of 1,109 BC patients and 113 normal paired tissues from the TCGA-BRCA (The Cancer Genome Atlas-Breast Cancer) program were enrolled in our study. The public RNA sequencing and clinical information of patients were obtained from TCGA^1^. The expression of 24 m6A-related genes was extracted from the TCGA-BRCA, including expression data on writers (METTL3, METTL14, METTL16, WTAP, VIRMA or KIA1499, RBM15, RBM15B, and ZC3H13), readers (YTHDC1, YTHDC2, YTHDF1, YTHDF2, YTHDF3, HNRNPC, HNRNPA2B1, IGF2BP1, IGF2BP2, IGF2BP3, FMR1, LRPPRC, and RBMX), and erasers (FTO and ALKBH5). The lncRNAs in the TCGA dataset were identified based on the annotation of Genome Reference Consortium Human Build 38 (GRCh38), and 14,086 lncRNAs were evaluated in the TCGA-BRCA transcriptome matrix. In this study, lncRNAs we identified were composed of eight types of transcripts (lincRNA, sense overlapping, retained intron, antisense, processed transcript, sense intronic, 3′ overlapping ncRNA, and non-coding and macro lncRNA). In this study, BC samples without complete survival data were excluded from all analyses. The clinical features of TCGA-BRCA samples are presented in Supplementary Table 1.

### Identification of N6-Methyladenosine-Related Long Non-coding RNAs

The Spearman correlation analysis was applied to screen m6A-related lncRNAs with the criteria of correlation coefficient > 0.4 and *p* < 0.001 in the TCGA-BRCA dataset. The network of m6A-related genes and m6A-related lncRNAs was visualized by Cytoscape software 3.5.1. To explore the prognostic value of m6A-related lncRNAs, univariate Cox regression analysis was conducted to select the m6A-related lncRNAs that were associated with patients’ overall survival (OS). The HR value and Cox *p* value were calculated. The Wilcoxon signed-rank test was used to compare the expression of m6A-related lncRNAs in BRCA tissues versus normal tissues.

### Breast Cancer Subtype Defined by N6-Methyladenosine-Related Long Non-coding RNAs

To systematically assess the roles and functions of m6A-related lncRNAs, cluster analysis was performed to subgroup BC patients into different groups using the ConsensusClusterPlus package of R software (Wilkerson and Hayes, 2010). Based on the expression level of prognostic-related lncRNAs, the consistent clustering algorithm was applied to determine the clustering number of samples. Then survival analysis was conducted to explore the prognostic value of different clusters. Comparison of clinical traits and different clusters was evaluated by the Chi-square test. The expression of checkpoint members including PDCD1 (PD-1), CD274 (PD-L1), CTLA4, LAG3, ICOS, and IDO1 was estimated in different BRCA clusters using the “limma” package.

### Construction of a Risk Model Based on N6-Methyladenosine-Related Long Non-coding RNAs

Based on univariate Cox regression, LASSO regression analysis was analyzed to explore the association between m6A-prognosis lncRNAs and BC risk. The TCGA BRCA patients were randomly divided into training cohort and validation cohort. Twenty-one m6A-related lncRNAs were selected to construct the best risk score model with the “glmnet” package. We calculated the riskScore of each sample with the formula: RiskScore = ExpressionLncRNA1 × CoefficientLncRNA1 + ExpressionLncRNA2 × CoefficientmLncRNA2 + … ExpressionLncRNAn × CoefficientLncRNAn. The samples were divided into high-risk and low-risk groups with the median value of the riskScore. Subsequently, Kaplan–Meier curves and receiver operating characteristic (ROC) curves of 1 year using “timeROC” package were adopted to evaluate the predictive accuracy of the risk model. In addition, univariate Cox regression analysis and multivariate Cox regression analysis were performed to assess the independence of the risk model by regarding risk score as a single characteristic factor. To evaluate the applicability of the model, we combined the clinical traits with riskScore to plot the survival curves.

### Evaluation of Immune Infiltration

To specifically analyze the immune infiltration, the CIBERSORT analytical tool was adopted to identify the difference of 22 types of immune cells in different BRCA clusters or high- and low-risk groups using the Wilcoxon rank-sum test (Newman et al., 2015). A comparison of the fraction of 22 immune cells in BRCA clusters was produced with “limma” packages in R. We performed ESTIMATE (Estimation of Stromal and Immune cells in MAlignant Tumors using Expression data) analysis to evaluate the level of immune cell infiltration, stromal level, and estimate score for each BRCA sample (Yoshihara et al., 2013). The immune, stromal, and estimate scores in different BRCA clusters were compared.

### Gene Set Enrichment Analysis

In our analysis, GSEA was used to determine the biological pathway exchanges in different BRCA clusters. The transcriptome expression data of TCGA-BRCA were analyzed. Gene sets with nominal *p* value < 0.05 were considered significant enrichments. Kyoto Encyclopedia of Genes and Genomes (KEGG) and Gene Ontology (GO) gene sets were performed with GSEA 4.1.0 tool.

### Statistical Analysis

All analyses were performed using R software (version 4.0.2). The Wilcoxon signed-rank test was adopted for two-group comparison, and the Kruskal–Wallis test was applied for comparison of more than two groups. With the best cutoff value for subgroup stratification, Kaplan–Meier curves were plotted to compare the OS among various subgroups and the log-rank *p* value was calculated. Univariate and multivariate Cox regression analyses were conducted to evaluate the independent prognostic value of the risk model based on m6A-related lncRNAs. *p*-value < 0.05 was considered statistically significant.

## Results

### Selection of N6-Methyladenosine-Related Long Non-coding RNAs in the Cancer Genome Atlas-Breast Cancer Cohort

**FIGURE 1:**
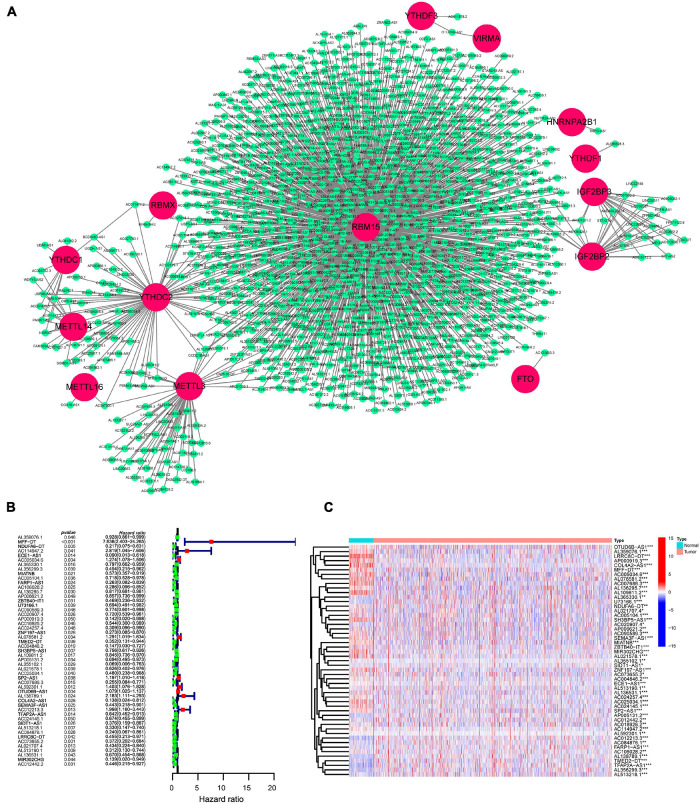
Identification of prognostic m6A-related lncRNAs in BC patients. **(A)** Construction of the m6A-regulator–lncRNA co-expression network using Cytoscape. Within networks, m6A-related genes are displayed in red nodes and m6A-related lncRNAs in green nodes. **(B)** A forest map showed 51 prognostic m6A-related lncRNAs identified by univariate Cox proportional hazard regression. **(C)** A heatmap displayed differentially expressed 51 m6A-related lncRNAs in the TCGA-BRCA dataset using the “limma” method. *p < 0.05; **p < 0.01; and ***p < 0.001.

We downloaded the RNA-Seq data of the TCGA-BRCA dataset and identified 14,086 lncRNAs using GRCh38 annotation. The expression of 24 m6A-related regulators was extracted, and then Spearman correlation analysis was performed. Using a threshold of correlation coefficient > 0.4 and *p* < 0.001, 491 lncRNAs were significantly correlated with m6A-related regulators and selected for further analysis. To directly visualize the correlation between lncRNAs and m6A-related regulators, a gene network was plotted by Cytoscape. The co-expression network of m6A-related regulators and lncRNAs showed that 14 m6A-related regulators (RBM15, RBMX, YTHDC1, YTHDC2, YTHDF1, YTHDF3, METTL3, METTL16, METTL14, IGF2BP2, IGF2BP3, HNRNRA2B1, VIRMA, and FTO) were significantly related to some lncRNAs, of which RBM15 was the hub regulator (Figure 1A). To further investigate the clinical significance of m6A-related lncRNAs, univariate Cox regression was performed. The forest plot displayed that 51 m6A-related lncRNAs were significantly associated with the OS of BC patients (Figure 1B). A heatmap plot compared the expression of 51 m6A-related lncRNAs for TCGA BC tissues versus normal tissues, and we found that all prognostic lncRNAs were abnormally expressed in BC (Figure 1C).

### Identification of N6-Methyladenosine-Related Long Non-coding RNAs and Immune Subtypes of Breast Cancer

**FIGURE 2:**
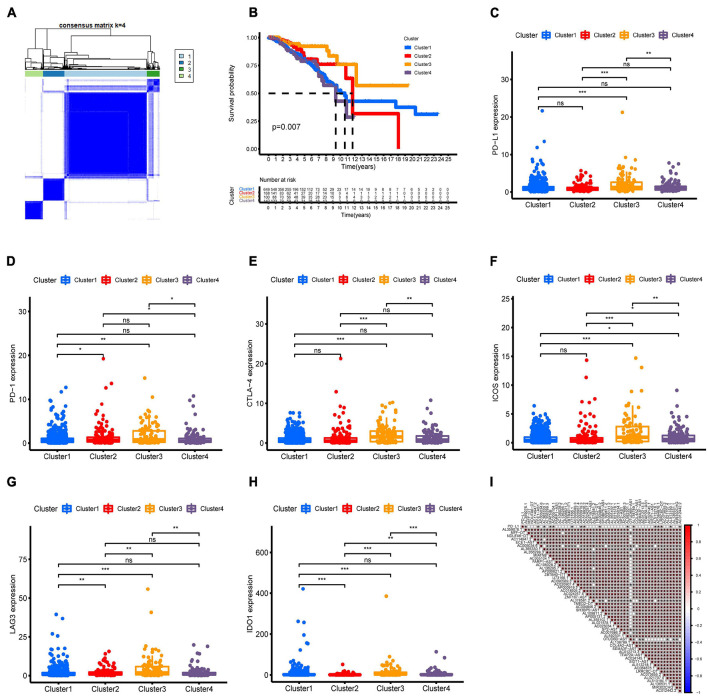
Consensus clustering of m6A-related lncRNAs and immune checkpoint member expression in different BC subtypes. **(A)** Consensus clustering matrix for k = 4. **(B)** Kaplan–Meier curves of OS for four clusters in TCGA-BRCA. (**C–H)** The expression of immune checkpoints PD-L1, PD-1, CTLA-4, ICOS, LAG3, and IDO1 in different BC subtypes. **(I)** Association of PD-L1 and 51 prognostic m6A-related lncRNAs was determined using Spearman correlation analysis. ns, no significance; *p < 0.05; **p < 0.01; and ***p < 0.001.

Molecular subtyping classification provides a fundamental basis for finding the optimal treatment for a particular patient. Therefore, we constructed consensus clustering analysis based on the expression profiles of 51 m6A-related prognostic lncRNAs. Intrigued by cumulative distribution, we chose k = 4 to subgroup BC patients where the sample cluster remained stable and robust (Figure 2A and Supplementary Figure 1). Kaplan–Meier analysis of OS revealed that Cluster 3 had the best prognosis, whereas Cluster 4 had the worst survival outcome (Figure 2B). We also explored the association of clinical characteristics with different BC clusters. The m6A-related lncRNA subgroup was only correlated with age at diagnosis (*p* < 0.05) (Supplementary Figure 2). Recently, immune therapies have evolved into a promising strategy for solid tumors. Therefore, we further estimated the expression of immune checkpoints in different BC clusters. As shown in Figure 2C, Cluster 3 patients displayed a significantly higher expression of PD-L1 compared to Cluster 1, Cluster 2, and Cluster 4. As expected, the same expression patterns of other immune checkpoint members including CTLA-4, PD-1, LAG3, and IDO1 in Cluster 3 were also observed (Figures 2D–H). Then, the correlation between PD-L1 and 51 m6A-related prognostic lncRNAs was analyzed. We observed a significant correlation between PD-L1 and 16 m6A-related lncRNAs (AL359076.1, ECE1-AS1, AL365330.1, MIATNB, AC005104.1, U73166.1, AP000919.3, AC018926.2, AC004846.2, AL592301.1, AL138789.1, AL513218.1, AC084876.1, LRRC8C-DT, AL513190.1, and AC012442.2) (Figure 2I). Moreover, the correlation of other immune checkpoint members and 51 m6A-related prognostic lncRNAs is presented in Supplementary Figure 3. The above findings indicated that BC patients with cluster 3 showed higher immunogenicity and might respond to the ICI therapy.

### Immune Landscape of N6-Methyladenosine-Related Long Non-coding RNAs Subtypes of Breast Cancer

**FIGURE 3:**
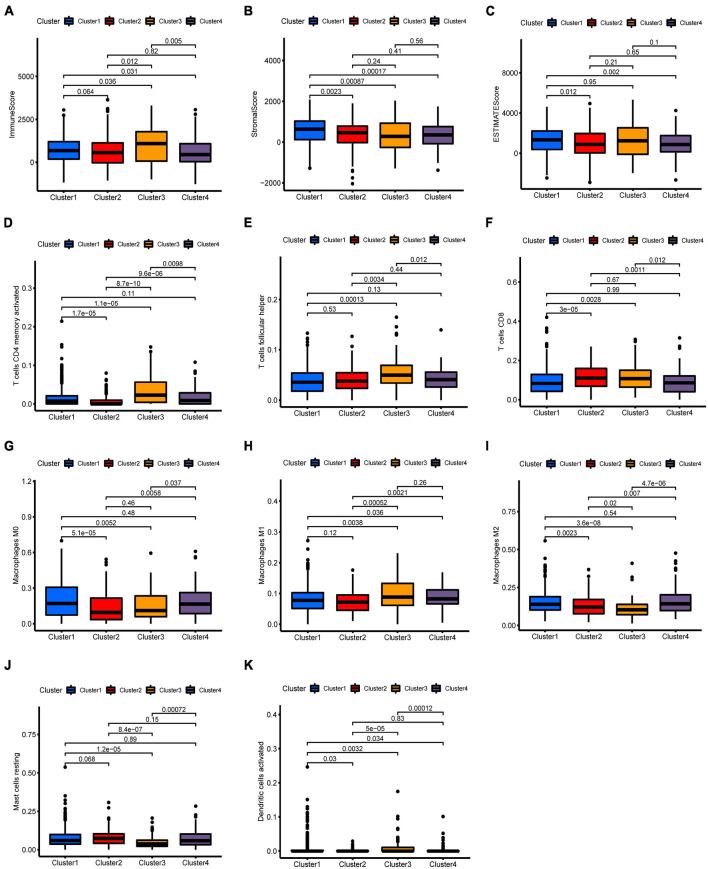
Immune characteristics among four m6A-related lncRNA BC clusters. **(A–C)** Different expression of immune score **(A)**, stromal score **(B)**, and ESTIMATE score **(C)** in four BC clusters. **(D–K)** Differences in the levels of infiltration of CD4 memory T cells, follicular helper T cells, CD8 T cells, M1 macrophages, M0 macrophages, M2 macrophages, resting mast cells, and dendritic cells in four BC clusters.

To thoroughly explore immune infiltration status in different BC clusters, CIBERSORT algorithms were used to assess the composition of the immune microenvironment. BC patients in cluster 3 tended to have a higher immune score and estimate score and a lower stroma score (Figures 3A–C). We further calculated individual immune infiltration in different BC clusters using the ESTIMATE program. Relative to other BC clusters, higher levels of activated CD4 memory T cells and follicular helper T cells were found in cluster 3 BC samples (Figures 3D,E). Cluster 3 BC samples also showed a higher infiltration of CD8 T cells compared to cluster 1 and cluster 4 (Figure 3F). High infiltration of M1 macrophages and low infiltration of M0 macrophages and M2 macrophages were observed in cluster 3 BC samples (Figures 3G–I). In addition, cluster 3 also presented the lowest level of resting mast cells and the highest level of dendritic cells among different BC clusters (Figures 3J,K). The above results suggested that cluster 3 might contain immune-hot tumors.

### Gene Set Enrichment Analysis

**FIGURE 4:**
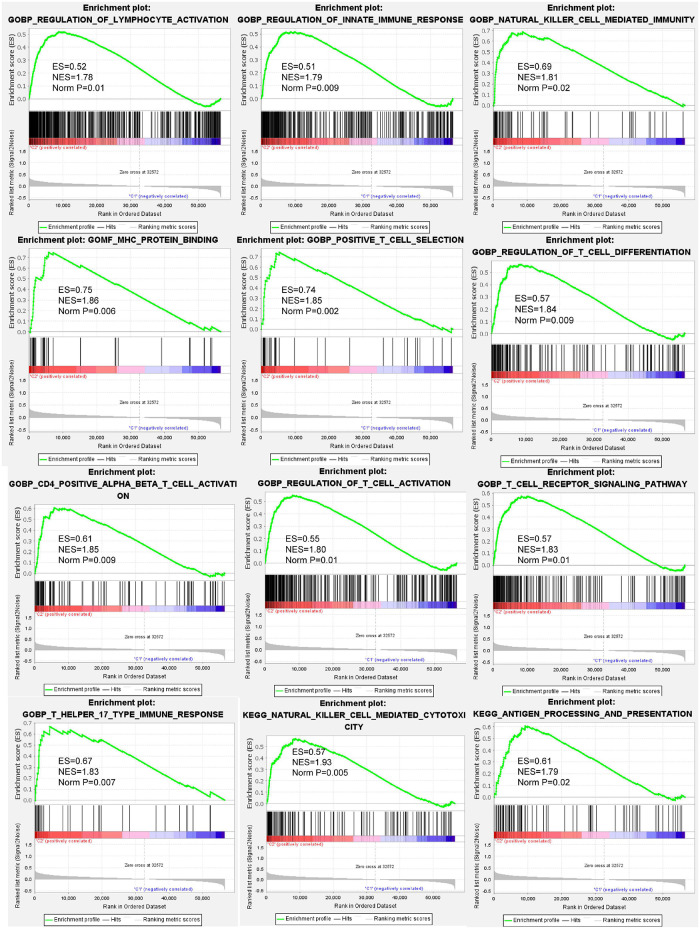
Gene set enrichment analysis (GSEA) indicating that various immune response processes were enriched in the BC cluster 3 patients compared to other BC subtypes. The above results demonstrated that biological processes such as lymphocyte activation, innate immune response, natural killer cell-mediated immunity, MHC protein binding, T cell activation, antigen processing and presentation, and so on were positively enrichment in BC cluster 3.

To annotate the potential biological process and pathways between cluster 3 and other clusters, GSEA analysis was computed using gene ontology (GO) and Kyoto Encyclopedia of Genes and Genomes (KEGG) signatures based on individual sample expression profiles. Our results showed that various immune response processes were significantly enriched in cluster 3 samples (Figure 4). GO signature revealed that MHC protein binding, natural cell-mediated immunity, T cell receptor signaling pathway, T cell activation, T help immune response, and so on were positively enriched in cluster 3. KEGG signature further revealed that cluster 3 was positively correlated with antigen processing and presentation and natural killer cell-mediated cytotoxicity.

### Generation of an N6-Methyladenosine-Related Long Non-coding RNAs Model for Prognosis

**FIGURE 5:**
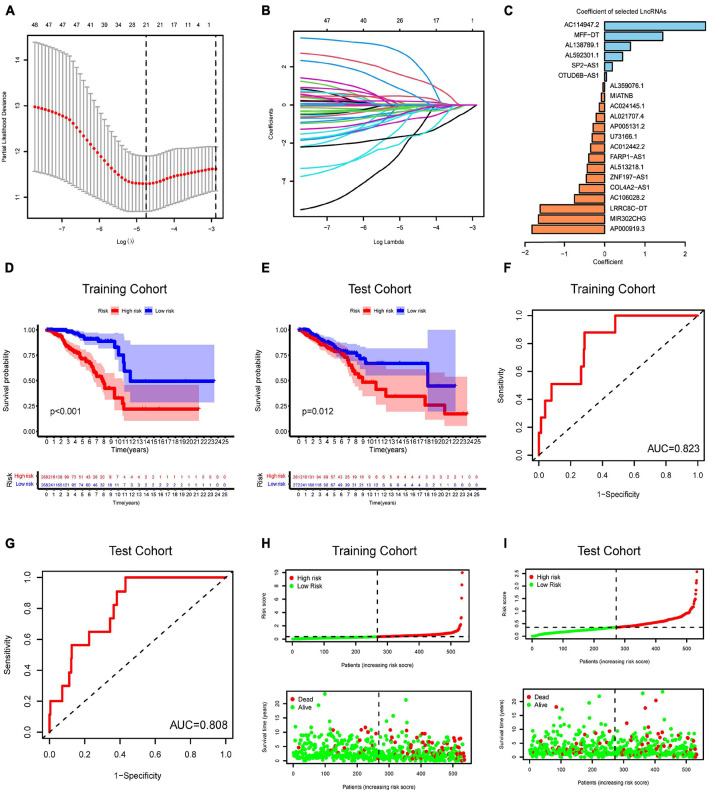
Risk model from m6A-related lncRNAs. **(A)** Elucidation for LASSO coefficient profiles of prognostic m6A-related lncRNAs. **(B)** The least absolute shrinkage was performed and selection operator (LASSO) regression model for OS. **(C)** Bar plot displayed the coefficients of 21 selected m6A-related lncRNAs. **(D,E)** Kaplan–Meier curves for OS of TCGA-BRCA patients based on the risk stratification in the training cohort **(D)** and test cohort **(E)**. **(F,G)** ROC analysis for OS prediction of TCGA-BRCA patients in the training cohort **(F)** and test cohort **(G)**. **(H,I)** Distribution of risk score and OS status in the TCGA training cohort **(H)** and TCGA test cohort **(I)**.

To construct an m6A-related lncRNA model for predicting the OS of BC patients, LASSO regression was performed based on the 51 m6A-related prognostic lncRNAs in the TCGA BRCA dataset. Twenty-one lncRNAs were identified as powerful prognostic lncRNAs *via* the LASSO analysis (Figures 5A,B). We then randomly divided BC patients into two sets: the training cohort (*N* = 536) and testing cohort (*N* = 533). The risk score for each group was calculated using the coefficient of 21 lncRNAs (Figure 5C). The detailed information of the 21 m6A-related lncRNAs for constructing the prognostic signature was presented in Table 1. According to the median risk score, each cohort was defined into high-risk group and low-risk group. Kaplan–Meier curves displayed that BC patients with higher risk scores had shorter OS than those with lower risk scores in the training cohort and testing cohort (Figures 5D,E). Moreover, ROC curves further demonstrated that m6A-LncRM had a promising prediction performance in the training cohort and testing cohort (Figures 5F,G). The ROC curves for 3 or 5 years were presented in Supplementary Figure 4. The risk score distribution and OS status for the training cohort was shown in Figure 5H. The scatter plots displayed the survival status of the training cohort and indicated that the death number of BC patients was increased with increasing risk score. Similar results were also observed in the testing cohort (Figure 5I). The expression heatmap of the 21 lncRNAs is shown in Supplementary Figure 5.

**TABLE 1:** The detailed information of the 21 m6A-related lncRNAs used to construct the prognostic signature.

**Gene symbol**	**Ensemble ID**	**HR**	**95% CI**	***p* value**	**Coefficient**
AL359076.1	ENSG00000236199	0.92	(0.86–0.99)	0.0464	–0.045041456857
MFF-DT	ENSG00000236432	7.63	(2.40–24.2)	0.0005	1.4424360252461
AC114947.2	ENSG00000261604	2.82	(1.04–7.60)	0.0406	2.5061147460099
MIATNB	ENSG00000244625	0.57	(0.35–0.91)	0.0207	–0.082370810408
FARP1-AS1	ENSG00000231194	0.26	(0.08–0.83)	0.0239	–0.395904051229
AC106028.2	ENSG00000258922	0.28	(0.09–0.85)	0.0245	–0.750480995586
U73166.1	ENSG00000230454	0.69	(0.49–0.98)	0.0388	–0.310333078783
AP000919.3	ENSG00000272625	0.14	(0.02–0.99)	0.0497	–1.807386283135
ZNF197-AS1	ENSG00000233509	0.27	(0.08–0.87)	0.0281	–0.457386210694
AP005131.2	ENSG00000267366	0.69	(0.49–0.97)	0.0340	–0.287258143512
SP2-AS1	ENSG00000234494	1.19	(1.00–1.41)	0.0384	0.1897165761445
AL592301.1	ENSG00000227512	1.40	(1.07–1.82)	0.0122	0.4433594581702
OTUD6B-AS1	ENSG00000253738	1.07	(1.02–1.13)	0.0039	0.0429010585291
AL138789.1	ENSG00000233589	2.18	(1.11–4.29)	0.0235	0.6389867346424
COL4A2-AS1	ENSG00000232814	0.13	(0.02–0.81)	0.0284	–0.631978531916
AC024145.1	ENSG00000255968	0.67	(0.45–0.99)	0.0496	–0.135908955676
AL513218.1	ENSG00000272100	0.32	(0.14–0.73)	0.0071	–0.434919309704
LRRC8C-DT	ENSG00000231999	0.45	(0.21–0.97)	0.0417	–1.603619279997
AL021707.4	ENSG00000230912	0.43	(0.22–0.84)	0.0132	–0.209802477279
MIR302CHG	ENSG00000249532	0.13	(0.02–0.94)	0.0441	–1.646526940502
AC012442.2	ENSG00000243389	0.44	(0.21–0.92)	0.0305	–0.350351658693

*HR, hazard ratio; CI, confidence interval.*

### Prognostic Value of the N6-Methyladenosine-Related Long Non-coding RNAs Model Signature

**FIGURE 6:**
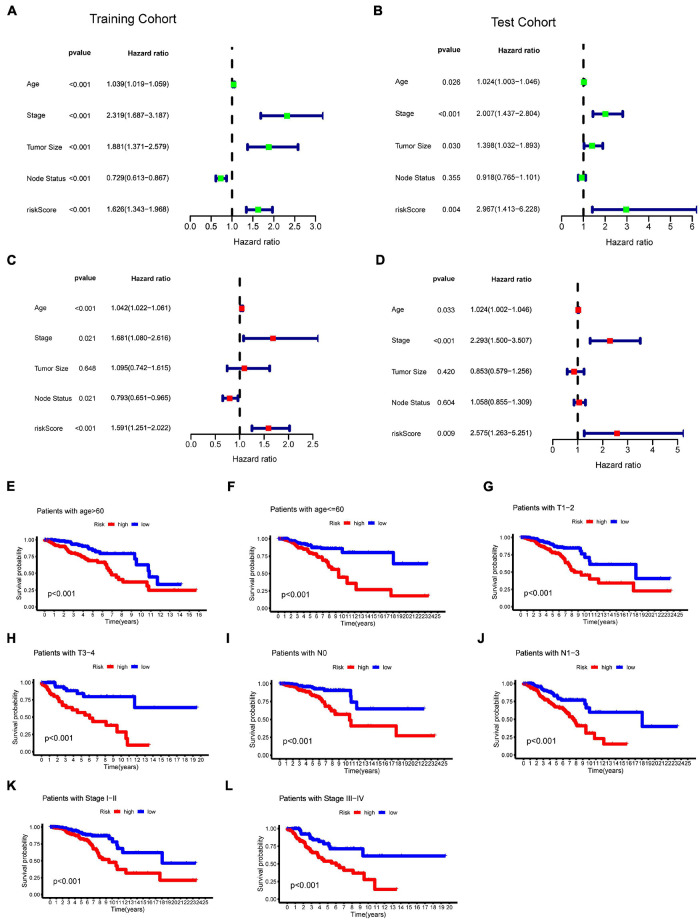
Evaluation of the prognostic role of m6ALncM signature. **(A,B)** The univariate Cox forest map of risk model score and clinical features in the TCGA training cohort **(A)** and TCGA test cohort **(B)**. **(C,D)** The multivariate Cox forest plot of risk model score and clinical characteristics in the training cohort **(C)** and test cohort **(D)**. **(E–L)** The m6A-LncRM signature retained its prognostic value in multiple subgroups of BC patients. Survival analysis in low- and high-risk groups adjusted by clinical factors including age, tumor size, node status, and tumor stage.

To evaluate whether the effect of m6A-LncRM on survival outcome was an independent risk factor for BC patients, we conducted univariate and multivariate Cox analyses for variables including age, tumor size, tumor stage, node status, and m6A-LncRM risk score. Univariable Cox regression analysis showed that m6A-LncRM was significantly correlated with OS of patients with BC [training cohort: HR = 1.626, 95% CI (1.343, 1.968), *p* < 0.001, Figure 6A; test cohort: HR = 2.967, 95% CI (1.413, 6.228), *p* = 0.004, Figure 6B]. Multivariate Cox analysis further indicated that m6A-LncRM remained an independent prognostic factor [training cohort: HR = 1.591, 95% CI (1.251, 2.022), *p* < 0.001, Figure 6C test cohort: HR = 2.575, 95% CI (1.263, 5.251), *p* = 0.009, Figure 6D]. In addition, clinical stratification analyses were performed after being adjusted by clinical factors including age, tumor size, node status, and tumor stage, and the results indicated that the high-risk group had worse OS than the low-risk group across all clinically stratified subgroups (Figures 6E–L).

### Relationship of the N6-Methyladenosine-Related Long Non-coding RNAs Model Signature and Clinical Features

**FIGURE 7:**
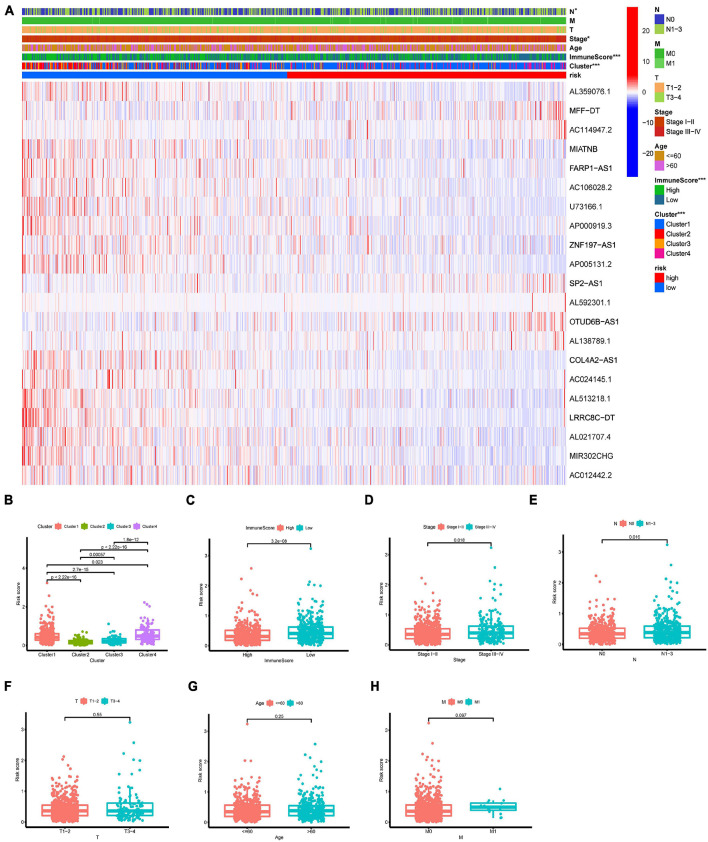
Clinical evaluation by the m6A-LncRM signature. **(A)** A strip heatmap along with the scatter diagram showed that clinical stage, BC clusters, and ImmuneScore were significantly associated with the riskScore. **(B–H)** BC patients with different clinicopathological features including m6A-LncRNA clusters, ImmuneScore, tumor stage, node status, tumor size, patient age, and metastasis status had different levels of risk scores.

We further investigated whether clinical features were associated with the m6A-LncRM risk score. Overall, the heatmap displayed that BC clusters, immuneScore, patients’ age, and node status between high- and low-risk groups were significantly different (Figure 7A). More specifically, the risk scores of cluster 2 and cluster 3 patients were significantly decreased relative to those of cluster 1 and cluster 4 (Figure 7B). BC patients with high immuneScore harbored significantly low risk scores (Figure 7C). Compared with stage I–II BC patients, patients with stage III–IV had higher risk scores (Figure 7D). BC patients with N1–N3 metastasis had higher risk scores versus the N0 group (Figure 7E). Moreover, metastatic disease also tended to have a higher risk score compared with that for BC patients without metastasis (Figure 7F). However, patients’ age and tumor size had no impact on risk scores (Figures 7G,H).

### Correlations Between N6-Methyladenosine-Related Long Non-coding RNAs Model Signature and Immune Cell Infiltration

**FIGURE 8:**
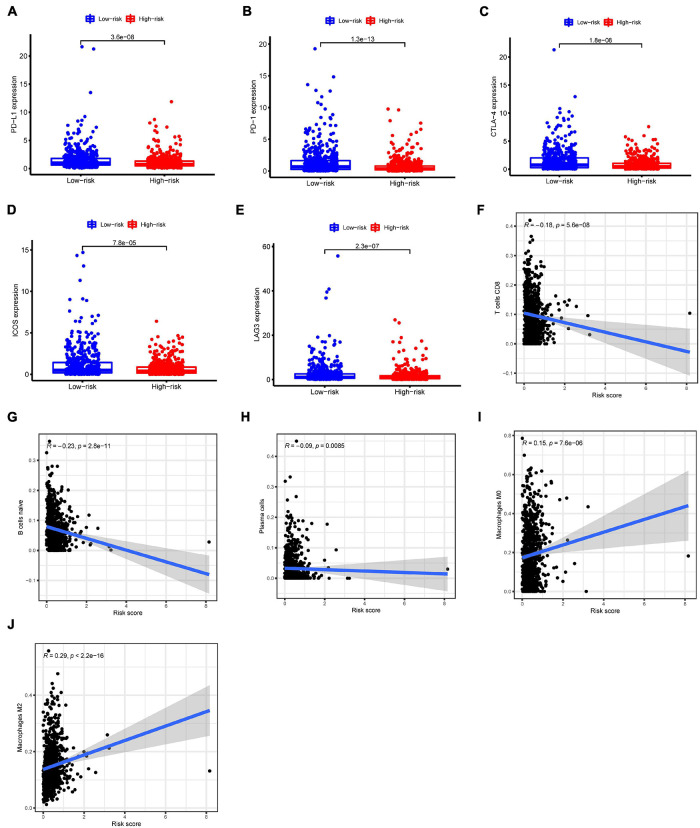
Relationship of immune cell infiltration and the m6A-LncRM signature. **(A–E)** The expression of immune checkpoint members including PD-L1, PD-1, CTLA-4, ICOS, and LAG3 in low-risk and high-risk BC groups. **(F–J)** m6ALncM signature negatively related to CD8 T cells, naive B cells, and plasma cells, while positive relationships were observed between m6ALncM and M0 macrophages and M2 macrophages.

We then investigated the differences of immune cell infiltration between high-risk and low-risk groups. Firstly, the expression of immune checkpoints was estimated. As shown in Figure 8A, low-risk BC patients displayed a significantly higher expression of PD-L1 compared to high-risk BC patients. The expression trend was nearly identical for other immune checkpoint members including PD-1, CTLA4, ICOS, and LAG3 (Figures 8B–E). CIBERSORT analysis showed that the risk score exhibited a negative relation to the infiltration of three types of immune cells: CD8 T cells (*r* = −0.18, *p* < 0.001), naive B cells (*r* = −0.23, *p* < 0.001), and plasma cells (*r* = −0.09, *p* = 0.008) (Figures 8F–H). The infiltration level of M0 macrophages and M2 macrophages was positively correlated with the risk score (Figures 8I,J).

## Discussion

In the current study, we aimed to identify the prognostic value of m6A-related lncRNAs. The expression of 24 m6A-related genes and 14,086 lncRNAs was extracted from the TCGA-BRCA cohort. Spearman correlation analysis and univariate Cox regression selected 51 m6A-related lncRNAs for further analysis. Consensus clustering analysis revealed a BC subtype (Cluster 3) with best prognosis, and a high expression of immune checkpoints was observed in Cluster 3. CIBERSORT and ESTIMATE analyses further revealed that a high level of immune cell infiltration was found in Cluster 3. GSEA analysis showed that cluster 3 samples were positively enriched in various immune response processes. Furthermore, 21 m6A-related lncRNAs were selected to establish m6A-LncRM for predicting the OS of BC patients. Survival analysis and ROC curves confirmed a promising prognostic value and prediction performance of m6ALncRM. Multivariate Cox regression analysis showed that m6A-LncRM was able to serve as an independent risk factor. Based on the median risk score, the high-risk group had worse survival outcome than the low-risk group across all clinically stratified subgroups. Finally, low- and high-risk BC subgroups displayed significantly different clinical features and immune cell infiltration status. In the present study, we identified a high immunogenicity BC subtype and developed a novel signature m6A-LncRM based on m6A-related lncRNA profiles of BC, which might serve as a predictive biomarker. Furthermore, we also developed a scoring system termed as m6A-LncRM risk score to evaluate individual m6A-related lncRNA patterns, which might be useful in identifying BC patients with high immunogenicity. Our study aimed to investigate the role of m6A in BC from a systems biology point of view, but not focusing on the mechanism of m6A modification in lncRNAs.

Existing classification methods of BC subtypes are restricted to mRNA expression analysis (Zhang et al., 2020). The PAM50 intrinsic subtypes of BC, based on the gene expression pattern, represents one of the most widely used protein-coding gene panels in clinical practice (Parker et al., 2009). Protein-coding transcripts merely represent about 2% of the human genome, while the rest are non-coding RNAs without protein-coding potential (Zhang et al., 2013). Recent bioinformatic research has identified an immune-related lncRNA signature model to predict the risk of 5-year recurrence-free survival in BC (Lai et al., 2020). Other studies have depicted the lncRNA landscape using transcriptome microarrays in BC and identified deregulated lncRNA expression patterns across different molecular subtypes (Cedro-Tanda et al., 2020). However, the overall perspective of m6A regulators involved in the dysregulation of lncRNAs in BC is not fully understood. As the most extensively studied RNA modification, m6A has been recognized as an epigenetic regulator affecting RNA splicing, stability, and translation. Currently, the field of lncRNA m6A modifications in BC is still in its relative infancy; thus, investigation of m6A-related lncRNAs from a “big data” perspective is warranted. LncRNAs could also recruit m6A regulators to exert their function. For instance, LINC00942 directly interacted with METTL14 protein by recognizing specific sequences, supporting its posttranscriptional m6A methylation modification of downstream targets in BC (Sun et al., 2020). Hypoxia-induced lncRNA KB-1980E6.3 was reported to recruit “readers” protein IGF2BP1 that enhanced c-Myc mRNA stability in BC (Zhu et al., 2021). The m6A modifications in lncRNAs were also widely observed. Recent findings indicated that METTL3-mediated m6A modification induced the high expression of LINC00958 by facilitating its RNA transcript stability in BC (Rong et al., 2021). The m6A modification of lncRNA Pvt1 was reported to participate in skin tissue homeostasis and wound repair (Lee et al., 2021). MeRIP-seq analysis detected multiple m6A sites of MALAT1, and its m6A residues could recruit YTHDC1 to nuclear speckles (Wang et al., 2021). In this study, we identified m6A-related lncRNAs using the correlation analysis and found that RBM15 had a complex network with numerous lncRNAs. The TCGA database and immunohistochemistry staining analysis indicated that RBM15 expression was upregulated in BC specimens (Liu et al., 2019). Up until now, no studies have yet analyzed its function and m6A-dependent mechanism in BC.

Since infiltration of various immune cells in the tumor microenvironment was observed in BC, BC was no longer considered as an immunological quiescent tumor type (Gil Del Alcazar et al., 2020). The advent of immunotherapy has attracted great attention of researchers and clinicians, which encompasses vaccines, oncolytic viruses, chimeric antigen receptor-modified T cells (CAR-T), relevant nanotechnology, and immune checkpoint blockade (Adams et al., 2019). Cancer vaccines have been under investigation in numerous ongoing clinical trials of BC such as dendritic cells, HER2-based vaccines, and peptide-targeting neoantigen vaccines (Hamilton et al., 2012; Fennemann et al., 2019). However, CAR-T cell therapy for BC is still in the early phase of clinical trials (Jin et al., 2020; Ferraro et al., 2021). Recent advances in nanotechnology allowed nanoparticles to co-deliver immunomodulatory agents that exhibited promising efficacy in BC (Li et al., 2021). Remarkably, the PD-1/PD-L1 monoclonal antibody was gradually coming to light and became a promising strategy for advanced TNBC patients (Schmid et al., 2020). Previously, BC was not believed to be an immunogenic tumor. Recent research progress has confirmed that some TNBC and HER2-positive subtypes were immunogenic (de Melo Gagliato et al., 2020). Numerous studies have reported that PD-L1 expression in breast cancer or inflammatory cells (Ghebeh et al., 2006; Joneja et al., 2017) and PD-L1 expression of cancer cells have long been suggested as a reliable indicator for anti-PD1/PD-L1 drugs (Reck et al., 2016). In our analysis, we identified a BC subtype that has a high expression abundance of immune checkpoint members, suggesting that this BC cluster might be suitable for immunotherapy approaches. To select most appropriate patients for ICI therapies, biomarkers that predict patients’ response to immunotherapy in BC are warranted. Infiltrating lymphocytes (TILs) have emerged as potential biomarkers of immunotherapy response. Increasing evidence revealed that the existence of TIL in the tumor microenvironment is strongly associated with better response rates for ICI therapies in “hot” or “inflamed” tumors (Rooney et al., 2015; Wein et al., 2017; Molinero et al., 2019). Apart from this, a high level of TIL infiltration also predicted a favorable response to neoadjuvant chemotherapy in BC (Wimberly et al., 2015). In this study, a higher level of TILs was found in cluster 3 BC samples relative to other BC clusters, which indicated that cluster 3 patients might benefit from ICI therapies. In concordance with immune infiltration, survival analysis also displayed a favorable outcome in cluster 3 patients. Finally, the GSEA results further suggested that immune-related biological processes were significantly enriched in cluster 3.

To improve the accuracy and efficacy on predicting outcome, we constructed a 21-m6A-related lncRNA model and found that this 21-lncRNA signature would independently predict OS in BC patients. Previous studies have attempted to identify an lncRNA-related panel for predicting the risk of tumor recurrence, neoadjuvant treatment response, and survival outcome (Guo et al., 2016; Wang et al., 2017; Li et al., 2018). Among the 21 candidate lncRNAs, only OTUD6B-AS1 and COL4A2-AS1 had been reported as prognostic factors in BC (Yao et al., 2019; Ma et al., 2020); the rest of lncRNAs were identified as prognostic signatures in BC for the first time. In the proposed model, we classified patients into low- and high-risk groups; we then reassessed the survival outcome, univariate and multivariate analyses, tumor immune infiltration, and expression of immune checkpoints. The low-risk group was associated with favorable survival, high level of TIL infiltration, high ImmuneScore, and high expression of immune checkpoint members, which indicated that this modeling algorithm functioned well.

Although m6A modifications of LncRNAs are still largely unexplored in BC, our analysis may provide the overall profile of m6A-related lncRNAs in BC. To our knowledge, this is the first report of the m6A-related lncRNA signature in BC. Undeniably, several drawbacks of our study should be noticed that needs to be further explored. Further *in vitro* and *in vivo* experiments are required to confirm the relationship of m6A-related regulators and lncRNA expressions. To explore the suitability and applicability of our model, more independent BC cohorts are needed to verify the prognostic value of the m6A-related lncRNAs model. Since the analyzed data were derived from databases, another limitation is that it is retrospective in nature. Additionally, the mechanism of m6A-related lncRNAs needs to be further investigated to improve the immunotherapy efficacy of BC.

## Conclusion

This study systematically explored the prognostic value of the m6A-related lncRNAs in BC. A high immunogenicity BC subtype was identified that could be a potential candidate for antitumor immunotherapy. The 21-lncRNA model is a robust biomarker that can independently predict OS of TCGA-BRCA datasets and immunotherapy efficacy.

## Data Availability Statement

The original contributions presented in the study are included in the article/Supplementary Material, further inquiries can be directed to the corresponding authors.

## Author Contributions

JZ performed most of the analyses and wrote the manuscript. XH and JC designed the study. BS, LL, JD, QS, and QZ performed some of the analyses and edited the manuscript. All authors contributed to the article and approved the submitted version.

## Conflict of Interest

The authors declare that the research was conducted in the absence of any commercial or financial relationships that could be construed as a potential conflict of interest.

## Publisher’s Note

All claims expressed in this article are solely those of the authors and do not necessarily represent those of their affiliated organizations, or those of the publisher, the editors and the reviewers. Any product that may be evaluated in this article, or claim that may be made by its manufacturer, is not guaranteed or endorsed by the publisher.

## Footnotes

1
https://portal.gdc.cancer.gov/
