# Corrigendum: Dissecting the Role of N6-Methylandenosine-Related Long Non-Coding RNAs Signature in Prognosis and Immune Microenvironment of Breast Cancer

**DOI:** 10.3389/fcell.2021.812770

**Published:** 2021-12-09

**Authors:** Jinguo Zhang, Benjie Shan, Lin Lin, Jie Dong, Qingqing Sun, Qiong Zhou, Jian Chen, Xinghua Han

**Affiliations:** Department of Medical Oncology, The First Affiliated Hospital of USTC, Division of Life Science and Medicine, University of Science and Technology of China, Hefei, China

**Keywords:** breast cancer, m6A-related lncRNAs, immune infiltration, gene signature, prognosis

In the published article, there was an error in affiliation **1**. Instead of “Division of Life Science and Medicine, Department of Medical Oncology, The First Affiliated Hospital of USTC, University of Science and Technology of China, Hefei, China,” it should be “Department of Medical Oncology, The First Affiliated Hospital of USTC, Division of Life Science and Medicine, University of Science and Technology of China, Hefei, China.”

The authors apologize for this error and state that this does not change the scientific conclusions of the article in any way. The original article has been updated.

