# Supplementary figures and images for: Dissecting the Role of N6-Methylandenosine-Related Long Non-coding RNAs Signature in Prognosis and Immune Microenvironment of Breast Cancer
Source: Front Cell Dev Biol. 2021 Oct 6;9:711859. doi: 10.3389/fcell.2021.711859 (PMC8526800; doi:10.3389/fcell.2021.711859)

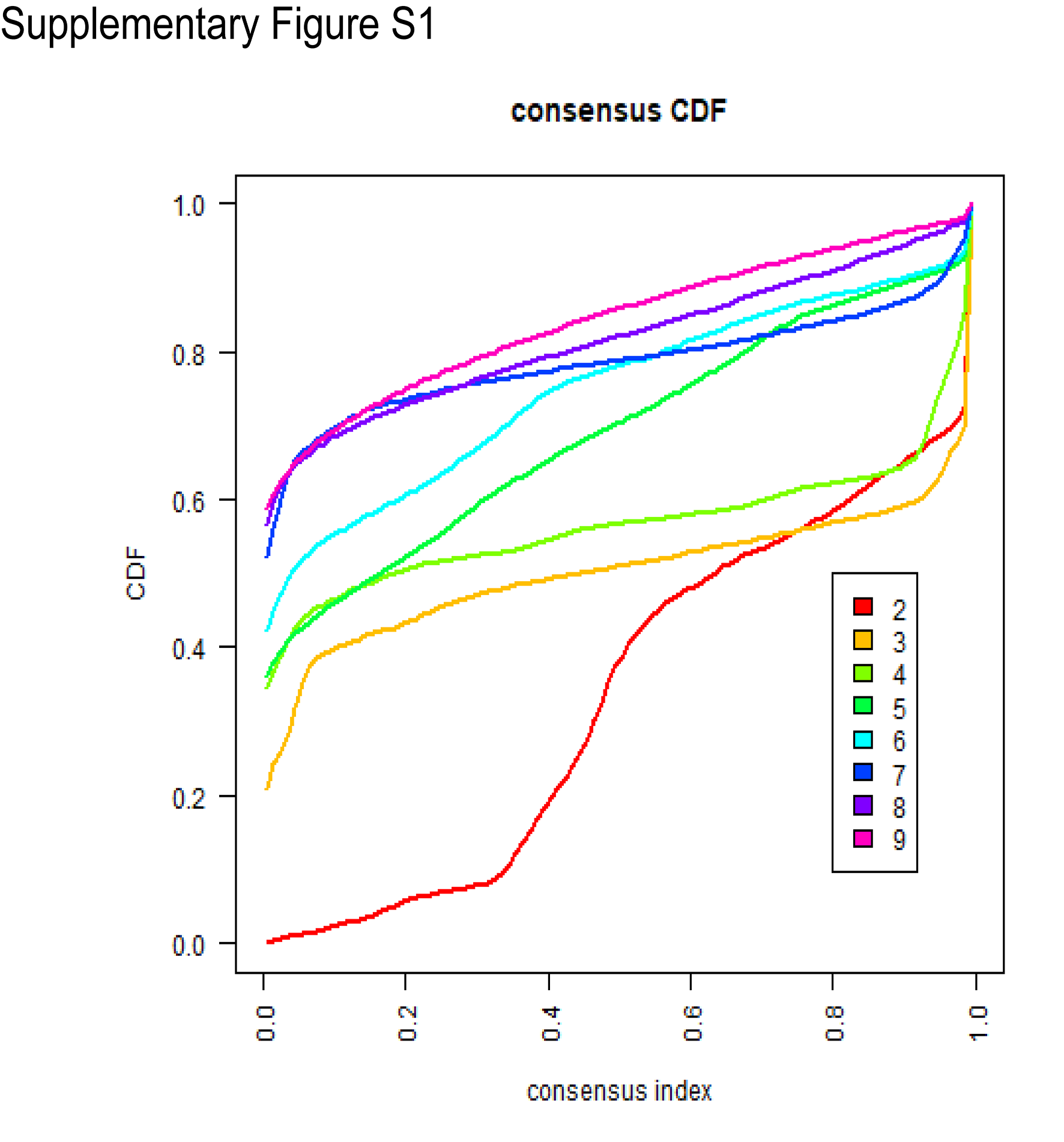

Supplement: Supplementary file 2 [file Image_1.TIF]

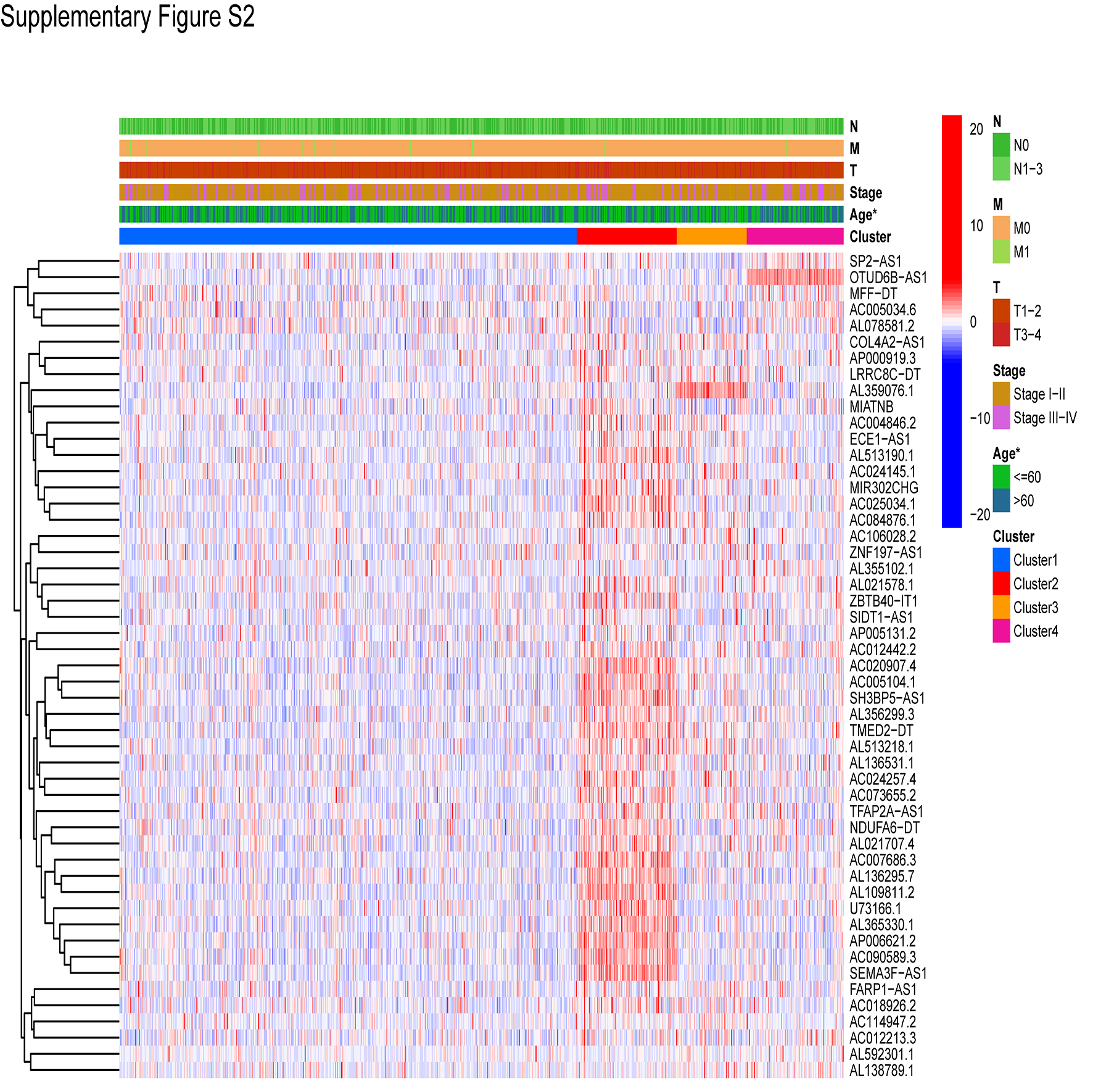

Supplement: Supplementary file 3 [file Image_2.TIF]

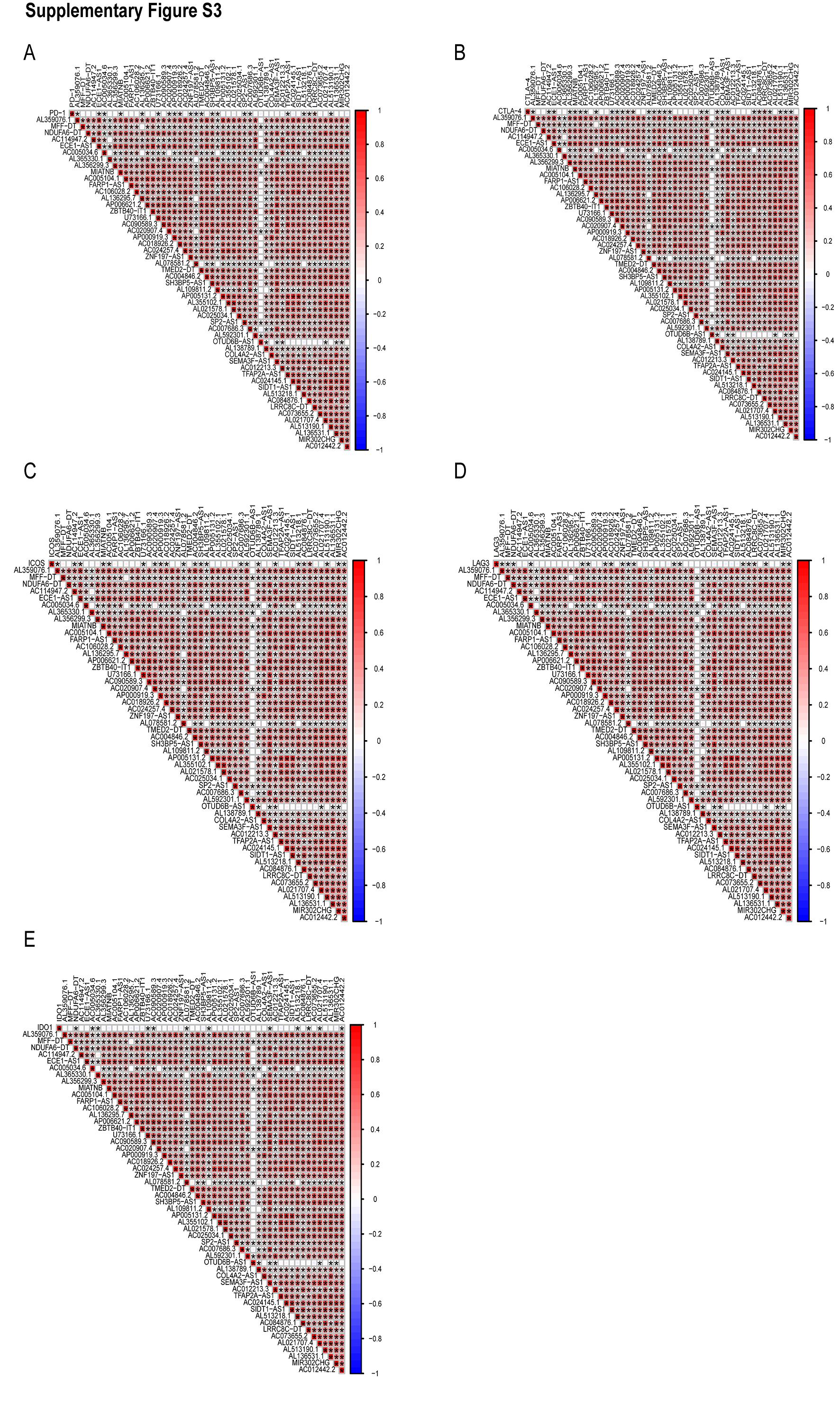

Supplement: Supplementary file 4 [file Image_3.TIF]

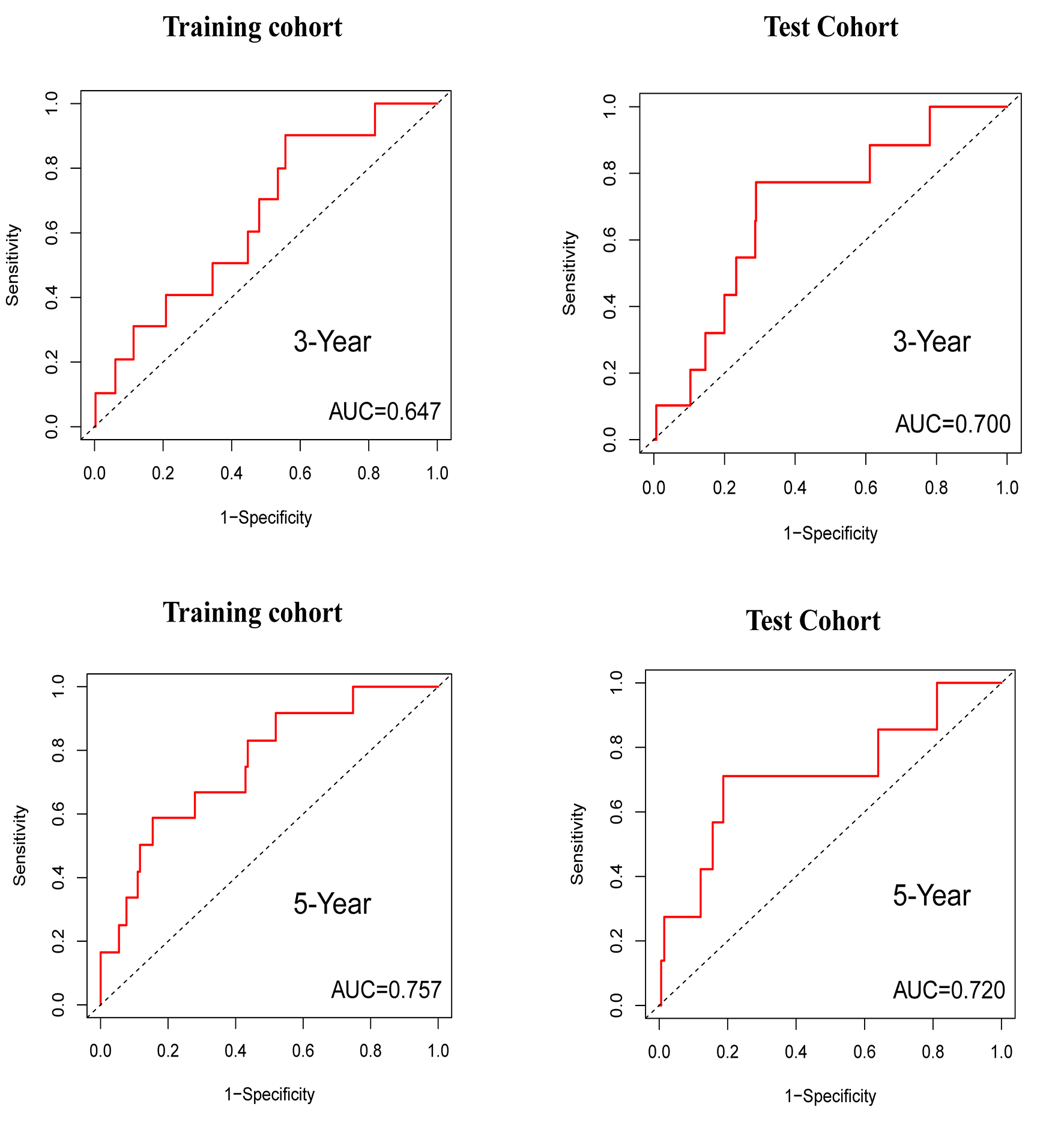

Supplement: Supplementary file 5 [file Image_4.TIF]

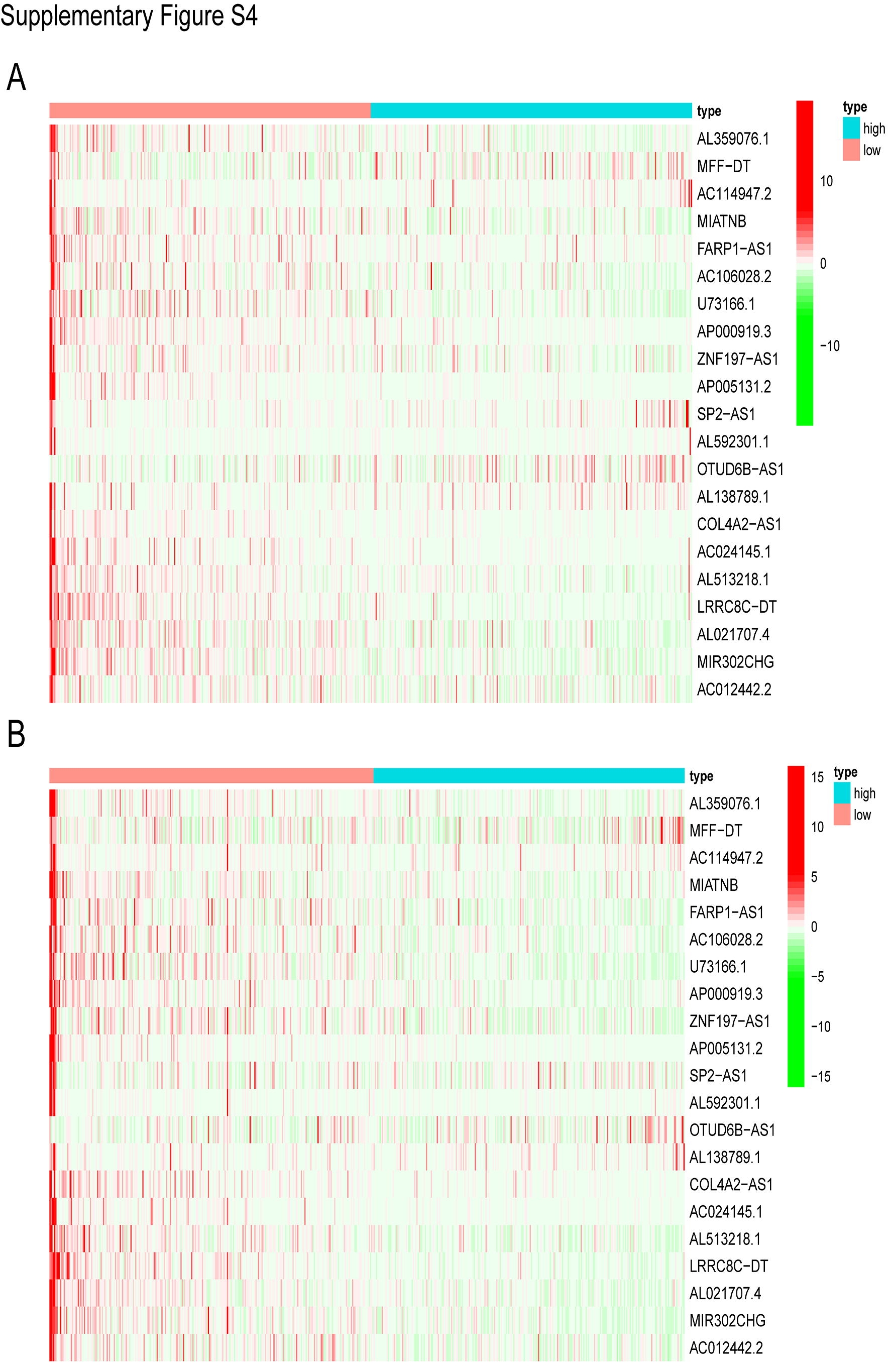

Supplement: Supplementary file 6 [file Image_5.TIF]
